# Basal pharmacokinetic parameters of topically applied diacerein in pediatric patients with generalized severe epidermolysis bullosa simplex

**DOI:** 10.1186/s13023-018-0940-1

**Published:** 2018-11-01

**Authors:** Michael Ablinger, Thomas K. Felder, Monika Wimmer, Roland Zauner, Peter Hofbauer, Thomas Lettner, Martin Wolkersdorfer, Florian B. Lagler, Anja Diem, Johann W. Bauer, Verena Wally

**Affiliations:** 1 EB House Austria, Research Program for Molecular Therapy of Genodermatoses, Department of Dermatology, University Hospital of the Paracelsus Medical University Salzburg, Muellner Hauptstrasse 48, 5020 Salzburg, Austria; 2 Department of Laboratory Medicine, Paracelsus Medical University, Salzburg, Austria; 3 Landesapotheke Salzburg, Department of Production, Hospital Pharmacy, Salzburg, Austria; 4 Institute for Inborn Errors of Metabolism and Department of Pediatrics, Paracelsus Medical University, Salzburg, Austria; 5 Department of Dermatology, University Hospital Salzburg of the Paracelsus Medical University Salzburg, Salzburg, Austria

**Keywords:** Epidermolysis bullosa, Diacerein, Topical application, Pharmacokinetics, Keratin

## Abstract

**Abstract:**

Generalized severe epidermolysis bullosa simplex (EBS-gen sev) is caused by mutations within either the *KRT5* or *KRT14* gene, phenotypically resulting in blistering and wounding of the skin and mucous membranes after minor mechanical friction. In a clinical phase 2/3 trial, diacerein has recently been shown to significantly reduce blister numbers upon topical application. In this study we addressed basic pharmacokinetic parameters of locally applied diacerein in vitro and in vivo.

Ex vivo experiments using a Franz diffusion cell confirmed the uptake and bio-transformation of diacerein to rhein in a porcine skin model. Rhein, the active metabolite of diacerein, was also detected in both urine and serum samples of two EBS-gen sev patients who topically applied a 1% diacerein ointment over a period of 4 weeks. The accumulated systemic levels of rhein in EBS-gen sev patients were lower than reported levels after oral application.

These preliminary findings point towards the uptake and prolonged persistance of diacerein / rhein within the intended target organ - the skin. Further, they imply an acceptable safety profile at the systemic level.

**Trial registration:**

DRKS. DRKS00005412. Registered 6 November 2013.

## Main text

Generalized severe epidermolysis bullosa simplex (EBS-gen sev) is caused by mutations within either the keratin 14 (*KRT14)* or keratin 5 (*KRT5)* gene, resulting in a susceptibility of the skin towards mechanical trauma. Due to the autosomal dominant mode of inheritance, conventional therapeutic approaches require high efficiency not only in generating sufficient amounts of a wild type allele, but also in replacing or down-regulating the disease causing copy. Although ex vivo gene therapy showed promising results in dystrophic and junctional subtypes of EB [1–3], these approaches are currently not applicable for dominatly inherited EBS. In addition to a small number of early-stage clinical trials or case reports on small molecule based treatment approaches for EBS [4], topically applied diacerein showed promising results in reducing blister numbers in two recent clinical studies [5, 6]. In vitro studies addressing the mode of action showed that diacerein, an antagonist of IL-1ß, reduced the aggregation of mutated keratin 14 (K14) and 5 (K5) protein upon heat shock, which ultimately leads to a disruption of the intermediate filament (IF) network, a characteristic observed for most EBS-gen sev underlying mutations in vitro [7]. This IF fragility not only leads to an increased expression and maturation of IL-1ß but also to an activation of the c-jun N-terminal kinase (JNK) stress pathway, which, in a positive feedback loop, promotes *KRT14* expression at increased levels [8]. In a pilot study, treatment of five EBS-gen sev patients demonstrated a positive effect of a 1% diacerein containing ointment on blister reduction. Blister numbers were reduced by more than 70% in treated skin areas and the reduction remained stable for 6 weeks [6]. In a phase 2/3 clinical trial, 17 patients topically applied a 1% diacerein cream or placebo once daily during a 4 week period onto 3% of their total body surface area (BSA), presenting with blisters at the start of the treatment. The outcome of this trial was a significant reduction of blister numbers in 60% of patients upon diacerein treatment within 4 weeks of application. At the end of a 3 months follow-up, 87% of diacerein-treated patients achieved this positive outcome, further substantiating the observation of a long-term effect of the treatment [5]. Despite the availability of pharmacokinetic data on orally administered diacerein, no such data regarding a topical application are currently available [9]. We therefore analyzed the metabolism of a 1% diacerein ointment both in vitro and in vivo in a volunteer extension of the phase 2/3 trial [5]*,* in order to verify the activation of the prodrug diacerein within the skin to support our understanding of rhein mediating the reduction in blister formation. In addition, we performed in vitro experiments using a Franz diffusion cell system with porcine skin as a surrogate for human skin to investigate whether or not deacetylation of the prodrug diacerein occurs within the skin.

**Fig. 1:**
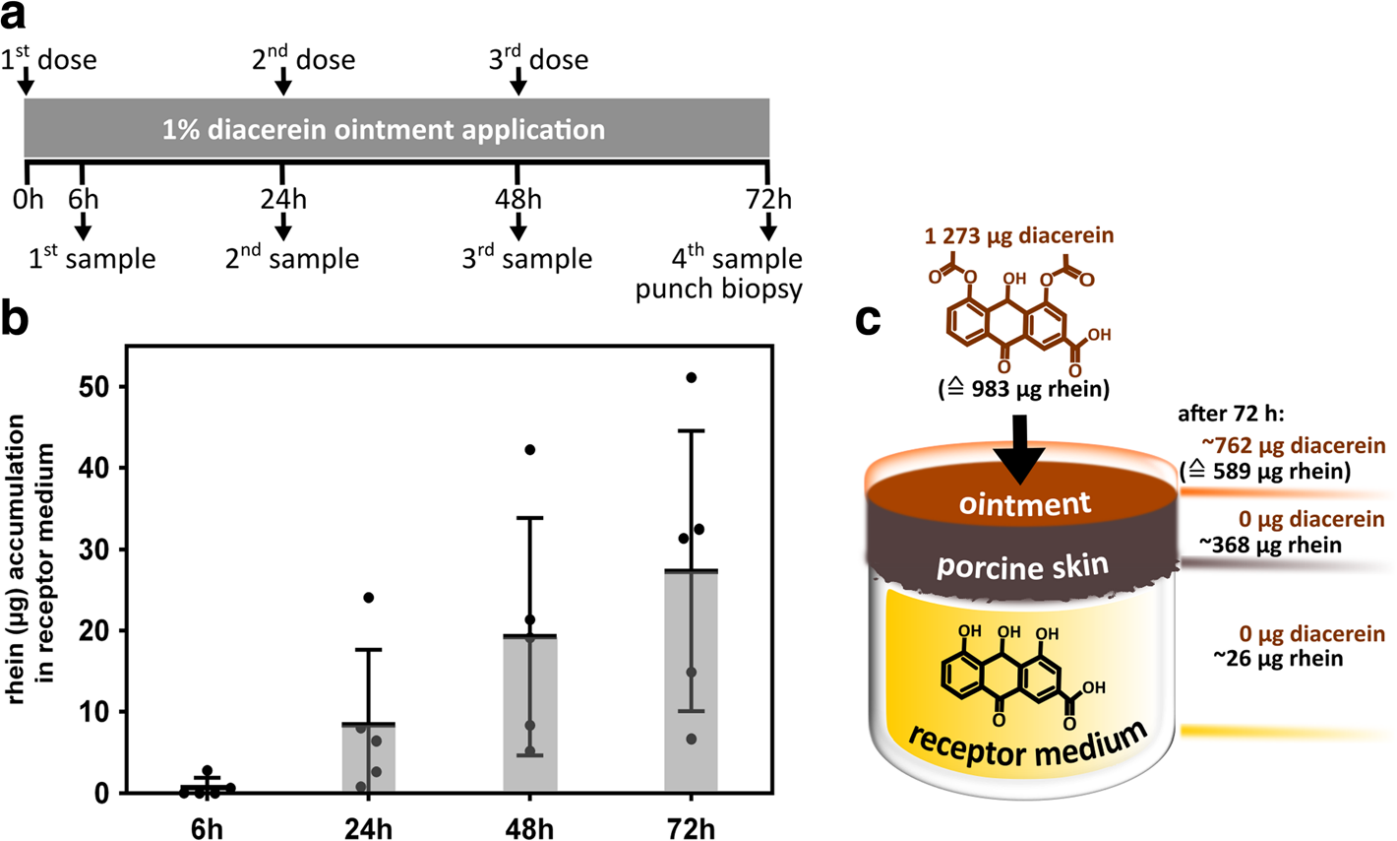
In vitro testing of diacerein pharmacokinetics. **a** Treatment regime of 1% diacerein ointment onto porcine skin model and sampling of Franz diffusion cell receptor medium and skin biopsy at final time point followed by mass-spectometry analysis of rhein levels. **b** Mass-spectrometric quantification of receptor medium samples showed accumulation of rhein over time. **c** After 72 h rhein was detected in both, receptor medium and skin biopsy, but no diacerein was detected at any time point. On average, approximately 40% of transformed rhein was retained within the skin (*n* = 5)

For that, skin samples (*n* = 5) were mounted on the 1cm^2^ Franz-cell and treated with a 1% diacerein ointment [10]. During a 72 h (hrs) time course, the 1% diacerein ointment was reapplied every 24 h and receptor medium was sampled for liquid chromatography tandem-mass spectrometry (LC-MS/MS) analysis after 6, 24, 48 and 72 h for evaluating the *trans*-epidermal permeation of diacerein / rhein [11]. In addition, 8 mm biopsies were taken from treated porcine skin at the end of the experiment, i.e. after 72 h, after thorough removal of any ointment remains, in order to determine rhein levels within the skin (Fig. 1a). After 6 h rhein was clearly detectable in the receptor medium in three out of five individual experiments (c_max_6hrs_ = 0.35 μg∙mL^− 1^). Continued drug application further increased rhein levels (at time points 24, 48, 72 h) with a c_max_72hrs_ of 6.39 μg/mL and a mean concentration c_mean_72hrs_ of 3.41 μg∙mL^− 1^ proving the transformation of diacerein into its active metabolite during skin permeation. In addition, we were also interested in the amount of rhein present within the skin after 72 h. On average 368 μg (SD = 85.7 μg) rhein were detected in the skin, meaning that 37.4% of the totally applied rhein, under the assumption of 100% conversion of diacerien to rhein, was retained within the skin after 72 h. Taking into account that 26 μg (SD = 17.1 μg, 2.7%) passed the skin, 589 μg (SD = 257.4 μg), representing 61.2%, of totally applied rhein (983 μg, SD = 276.6 μg) remained within the acceptor compartment (Fig. 1b, c). As only rhein, but not diacerein, was detected in both, receptor medium and skin biopsy, we conclude that diacerein is rapidly metabolized within the skin into its active form rhein, relevant for the therapeutic strategy in treating EBS-gen sev patients.

**Fig. 2:**
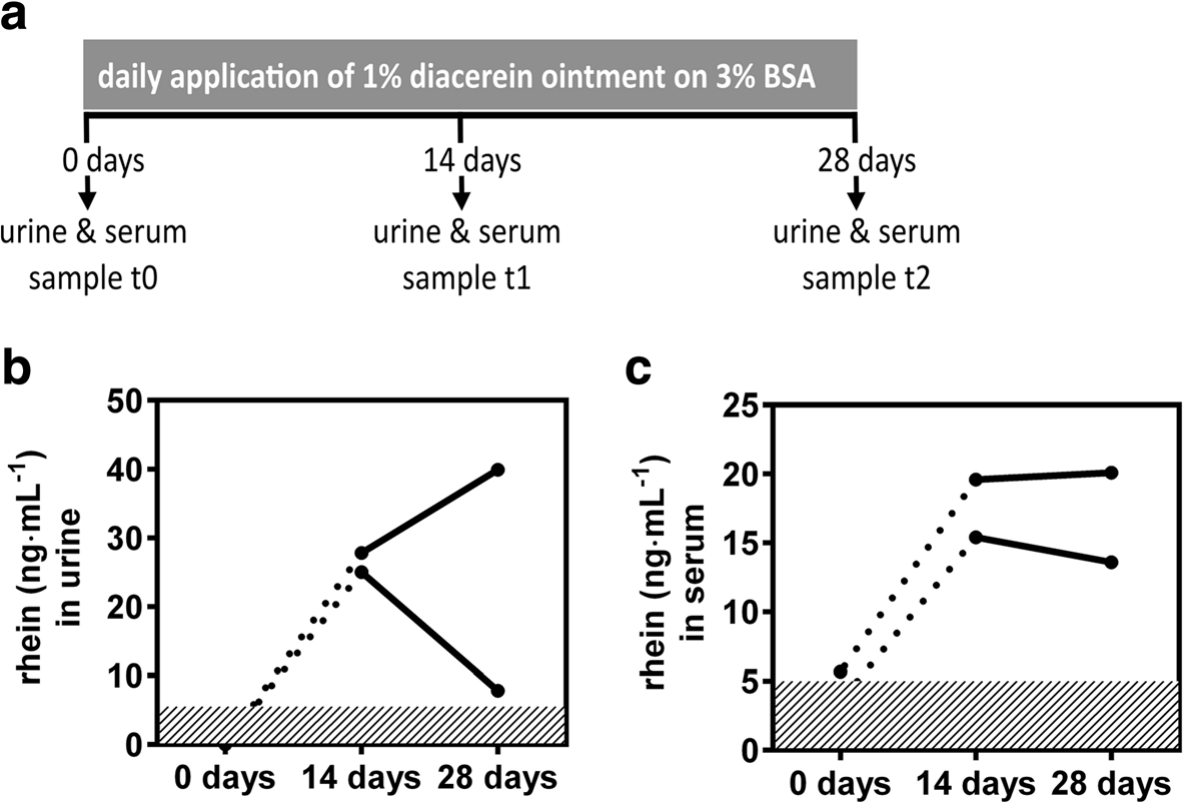
In vivo pharmacokinetics of diacerein derived rhein. **a** Experimental outline and LC-MS/MS analysis of **b** urine and **c** serum samples: rhein serum levels remained stable; fluctuations are evident in urine, pointing towards inter-individual variations. Urine samples were normalized to creatinine

In addition to the ability of skin to convert diacerein, we were interested in pharmacokinetics in vivo to assess systemic rhein levels. EBS-gen sev patients, who had participated in the clinical phase 2/3 diacerein trial, topically applied the 1% ointment over a period of four weeks onto 3% of their body surface areas (BSA) in a volunteer pharmacokinetic extension study of the clinical trial [5] (Fig. 2a). Given the burden of children with EBS-gen sev, only 2 patients were willing to participate in this pharmacokinetic (PK)-trial. BSA for patient 1 was a 310 cm^2^ area on the right thigh and a 210 cm^2^ area stretching from the left thigh into the left groin for patient 2, both presenting with blisters at the start of the treatment. In total, 123.4 g and 69.9 g of 1% diacerein cream, respectively, were applied, amounting for a calculated, average daily dose of 34 mg rhein, under the assumption of complete conversion of diacerein, for patient 1 and 19 mg rhein for patient 2. To evaluate systemic absorption upon topical application, blood and urine samples were obtained when starting the treatment, and after 14 and 28 days. Rhein was detected in all samples from both patients. In patient 1, maximum serum levels of c_max_serum_ = 20.1 ng∙mL^− 1^ and creatinine normalized maximum urine levels c_max_urine_ of 39.9 ng∙mL^− 1^ were measured. In patient 2, 15.4 ng∙mL^− 1^ in serum and a c_max_urine_ = 25.0 ng∙mL^− 1^ in urine were detected at maximum (Fig. 2b, c, Table 1). While serum levels remained rather stable, rhein levels differed significantly between patients after 4 weeks of treatment, potentially pointing towards differences in renal clearance, which will need to be taken into account in future studies.

**Table 1:** Baseline characteristics

Patient number	Age (years)	Sex	Total amount of cream used (g)	Rhein mean daily dose (mg)	Total area treated (cm^2^)	c_max_urine_ (ng∙mL^−1^)	c_max_serum_ (ng∙mL^− 1^)
1	8	m	123.4	34	310	39.9	20.1
2	7	m	69.9	19	210	25.0	15.4

Two patients who applied 1% diacerein for a period of four weeks on 3% BSA were included in the pharmacokinetic analysis. Serum and urine samples were taken every two weeks. Detected maximum levels (c_max_) of rhein in the urine and serum are given

In conclusion, given our results and comparing them to already published data on oral administration by Nicolas et al., treatment of 3% of the body surface for 4 weeks resulted in systemic rhein levels that were approximately 150-fold lower than the levels detected 24 h after single-dose oral intake. A maximum of 10.23 mg total rhein in the plasma was determined upon oral administration of a 50 mg single dose diacerein [9] . Even when extrapolating our data from 3% BSA (rhein levels in serum: 20.1 ng∙mL^− 1^) up to a treatment of 90% BSA (603 ng∙mL^− 1^) – which relates to covering the whole body except head and genitals – reported levels measured upon oral administration (9100 ng∙mL^− 1^) would not be reached. As an anthraquinone derivative, oral administration of diacerein has been reported to cause major side effects affecting the gastro-intestinal tract, so that the European Medicinal Agency (EMA) no longer recommends its use in patients aged 65 years and older. However, topical application of diacerein renders the probability of such side effects highly unlikely.

Despite several attempts using both RNA and genome editing techniques to restore wild type *KRT14* and *KRT5*, no causal therapy for EBS-gen sev is currently available to treat patients [12–14]. Therefore, treatments to reduce characteristic skin manifestations, thereby increasing patient’s quality of life are urgently needed and small molecules could provide a remedy. A few such approaches for different EB subtypes have been published during the last years, most of them being small clinical trials or case reports [15–22]. For EBS however, none of these studies has reached the level of late phase clinical trials yet [23–27].

In order to reduce blister number and increase EBS-gen sev patient’s quality of life, the anti-inflammatory effect of diacerein was investigated in a recent phase 2/3 clinical trial, which showed promising results that provided the basis for a worldwide phase III clinical trial (NCT03154333) [5]. Knowledge about basal pharmacokinetics will provide important information regarding the safety of the ointment.

In summary, our results demonstrate that the prodrug diacerein is metabolized to its active form rhein within the skin, thereby allowing for the exertion of its anti-inflammatory effect in EBS-gen sev patient skin. In vivo, patients showed no side effects or complications related to the ointment over the time course of the treatment matching the results of two clinical trials on EBS-gen sev including 22 patients in total, where no treatment-related side effects were reported [5, 6]. However, there are some major limitations of this study, especially as in vivo data is limited to only two young test subjects. Given that the patient cohort included in this study are children who suffer from skin lesions and impaired wound healing, blood sampling was not compulsory as part of the previous phase 2/3 clinical trial. This would have drastically reduced patients’ willingness to participate in the study, which would have potentially caused recruitment failure in this particularly rare disease. Indeed, this is a major problem we face in many EB trials and in rare (pediatric) diseases in general. Nevertheless, we believe that preliminary data on PK are important in order to provide the basis for more extensive PK studies that are necessary for drug development. Notably, based on such results, patient numbers for PK sampling can be properly calculated, potentially reducing the number of patients to be included.

Finally, we propose that 1% diacerein ointment is a safe and well-tolerated targeted therapy for the treatment of epidermolysis bullosa.

## Abbreviations

BSA: Body surface area
EB: Epidermolysis bullosa
EBS-gen sev: Generalized severe epidermolysis bullosa simplex
EMA: European Medicinal Agency
IF: Intermediate filament
JNK pathway: c-jun N-terminal kinase stress pathway
K: Keratin (protein)
KRT: Keratin (gene)
LC-MS/MS: Liquid chromatography tandem-mass spectrometry
PK: Pharmacokinetic

## References

[1] Mavilio F, Pellegrini G, Ferrari S, Di Nunzio F, Di Iorio E, Recchia A (2006). Correction of junctional epidermolysis bullosa by transplantation of genetically modified epidermal stem cells. Nat Med.

[2] Siprashvili Z, Nguyen NT, Gorell ES, Loutit K, Khuu P, Furukawa LK (2016). Safety and wound outcomes following genetically corrected autologous epidermal grafts in patients with recessive dystrophic epidermolysis bullosa. JAMA.

[3] Hirsch T, Rothoeft T, Teig N, Bauer JW, Pellegrini G, De Rosa L (2017). Regeneration of the entire human epidermis using transgenic stem cells. Nature.

[4] Pfendner EG, Bruckner AL, Adam MP, Ardinger HH, Pagon RA, Wallace SE, LJH B, Stephens K, Amemiya A (1993). Epidermolysis Bullosa Simplex.

[5] Wally V, Hovnanian A, Ly J, Buckova H, Brunner V, Lettner T (2018). Diacerein orphan drug development for epidermolysis bullosa simplex: a phase 2/3 randomized, placebo-controlled, double-blind clinical trial. J Am Acad Dermatol.

[6] Wally V, Kitzmueller S, Lagler F, Moder A, Hitzl W, Wolkersdorfer M (2013). Topical diacerein for epidermolysis bullosa: a randomized controlled pilot study. Orphanet J Rare Dis..

[7] Chamcheu JC, Siddiqui IA, Syed DN, Adhami VM, Liovic M, Mukhtar H (2011). Keratin gene mutations in disorders of human skin and its appendages. Arch Biochem Biophys.

[8] Wally V, Lettner T, Peking P, Peckl-Schmid D, Murauer EM, Hainzl S (2013). The pathogenetic role of IL-1beta in severe epidermolysis bullosa simplex. J Invest Dermatol.

[9] Nicolas P, Tod M, Padoin C, Petitjean O (1998). Clinical pharmacokinetics of diacerein. Clin Pharmacokinet.

[10] Bartosova L, Bajgar J (2012). Transdermal drug delivery in vitro using diffusion cells. Curr Med Chem.

[11] Layek B, Kumar TS, Trivedi RK, Mullangi R, Srinivas NR (2008). Development and validation of a sensitive LC-MS/MS method with electrospray ionization for quantitation of rhein in human plasma: application to a pharmacokinetic study. Biomed Chromatogr.

[12] Liemberger B, Pinon Hofbauer J, Wally V, Arzt C, Hainzl S, Kocher T (2018). RNA trans-splicing modulation via antisense molecule interference. Int J Mol Sci.

[13] Peking P, Breitenbach JS, Ablinger M, Muss WH, Poetschke FJ, Kocher T et al. An ex-vivo RNA trans-splicing strategy to correct human generalized severe epidermolysis bullosa simplex. Br J Dermatol. 2018. doi: 10.1111/bjd.17075. 10.1111/bjd.17075 PMC6334280 30099737

[14] Aushev M, Koller U, Mussolino C, Cathomen T, Reichelt J (2017). Traceless targeting and isolation of gene-edited immortalized keratinocytes from epidermolysis bullosa simplex patients. Mol Ther Methods Clin Dev.

[15] Humbert P, Renaud A, Laurent R, Agache P (1989). Tetracyclines for dystrophic epidermolysis bullosa. Lancet.

[16] Ozanic Bulic S, Fassihi H, Mellerio JE, McGrath JA, Atherton DJ (2005). Thalidomide in the management of epidermolysis bullosa pruriginosa. Br J Dermatol.

[17] Fine JD, Manes B, Frangoul H (2015). Systemic granulocyte colony-stimulating factor (G-CSF) enhances wound healing in dystrophic epidermolysis bullosa (DEB): results of a pilot trial. J Am Acad Dermatol.

[18] Chiaverini C, Fontas E, Vabres P, Bessis D, Mazereeuw J, Charlesworth A (2015). Oral erythromycin therapy in epidermolysis bullosa simplex generalized severe. Br J Dermatol.

[19] Chiaverini C, Passeron T, Lacour JP (2016). Topical timolol for chronic wounds in patients with junctional epidermolysis bullosa. J Am Acad Dermatol.

[20] Chiaverini C, Roger C, Fontas E, Bourrat E, Bourdon-Lanoy E, Labreze C (2016). Oral epigallocatechin-3-gallate for treatment of dystrophic epidermolysis bullosa: a multicentre, randomized, crossover, double-blind, placebo-controlled clinical trial. Orphanet J Rare Dis.

[21] Bornert O, Peking P, Bremer J, Koller U, van den Akker PC, Aartsma-Rus A (2017). RNA-based therapies for genodermatoses. Exp Dermatol.

[22] Nystrom A, Bernasconi R, Bornert O (2018). Therapies for genetic extracellular matrix diseases of the skin. Matrix Biol.

[23] Kerns ML, Guss L, Fahey J, Cohen B, Hakim JM, Sung S (2017). Randomized, split-body, single-blinded clinical trial of topical broccoli sprout extract: assessing the feasibility of its use in keratin-based disorders. J Am Acad Dermatol.

[24] Weiner M, Stein A, Cash S, de Leoz J, Fine JD (2004). Tetracycline and epidermolysis bullosa simplex: a double-blind, placebo-controlled, crossover randomized clinical trial. Br J Dermatol.

[25] Veien NK, Buus SK (2000). Treatment of epidermolysis bullosa simplex (EBS) with tetracycline. Arch Dermatol.

[26] Retief CR, Malkinson FD, Pearson RW (1999). Two familial cases of epidermolysis bullosa simplex successfully treated with tetracycline. Arch Dermatol.

[27] Abitbol RJ, Zhou LH (2009). Treatment of epidermolysis bullosa simplex, weber-Cockayne type, with botulinum toxin type a. Arch Dermatol.

